# Integration of 3D-printed middle ear models and middle ear prostheses in otosurgical training

**DOI:** 10.1186/s12909-024-05436-9

**Published:** 2024-04-24

**Authors:** Sini Lähde, Yasmin Hirsi, Mika Salmi, Antti Mäkitie, Saku T. Sinkkonen

**Affiliations:** 1 Department of Otorhinolaryngology – Head and Neck Surgery, Head and Neck Center Tauno Palva Laboratory, Helsinki University Hospital and University of Helsinki, Helsinki, Finland; 2 King’s College London, London, UK; 3 Department of Mechanical Engineering, Aalto University, Espoo, Finland; 4 Faculty of Medicine, Research Program in Systems Oncology, University of Helsinki, Helsinki, Finland

**Keywords:** Middle ear, Ossiculoplasty, Ossicles, 3D printing, Temporal bone, PORP

## Abstract

**Background:**

In otosurgical training, cadaveric temporal bones are primarily used to provide a realistic tactile experience. However, using cadaveric temporal bones is challenging due to their limited availability, high cost, and potential for infection. Utilizing current three-dimensional (3D) technologies could overcome the limitations associated with cadaveric bones. This study focused on how a 3D-printed middle ear model can be used in otosurgical training.

**Methods:**

A cadaveric temporal bone was imaged using microcomputed tomography (micro-CT) to generate a 3D model of the middle ear. The final model was printed from transparent photopolymers using a laser-based 3D printer (vat photopolymerization), yielding a 3D-printed phantom of the external ear canal and middle ear. The feasibility of this phantom for otosurgical training was evaluated through an ossiculoplasty simulation involving ten otosurgeons and ten otolaryngology–head and neck surgery (ORL-HNS) residents. The participants were tasked with drilling, scooping, and placing a 3D-printed partial ossicular replacement prosthesis (PORP). Following the simulation, a questionnaire was used to collect the participants' opinions and feedback.

**Results:**

A transparent photopolymer was deemed suitable for both the middle ear phantom and PORP. The printing procedure was precise, and the anatomical landmarks were recognizable. Based on the evaluations, the phantom had realistic maneuverability, although the haptic feedback during drilling and scooping received some criticism from ORL-HNS residents. Both otosurgeons and ORL-HNS residents were optimistic about the application of these 3D-printed models as training tools.

**Conclusions:**

The 3D-printed middle ear phantom and PORP used in this study can be used for low-threshold training in the future. The integration of 3D-printed models in conventional otosurgical training holds significant promise.

**Supplementary Information:**

The online version contains supplementary material available at 10.1186/s12909-024-05436-9.

## Background

Anatomically, the temporal bone is one of the most complex parts of the human body [1, 2]. The precise relationship and sensitivity of its structures make temporal bone surgery challenging but necessary for surgical training. The integration of virtual simulations, three-dimensional (3D) printed models, and hybrid training methods are reshaping otosurgery education, benefiting both trainees and patients [3].

Traditional training using cadaveric temporal bones provides the most realistic experience of otosurgical procedures [4]. However, the use of cadaveric temporal bones presents challenges, such as limited availability, high costs, potential infection hazards, and ethical considerations. Some of these challenges can be avoided by using animal temporal bones. However, in this case, other issues arise, such as anatomical differences from human bone, as well as the need for dedicated laboratory facilities [4]. Another alternative are virtual models such as Visible Ear® [5] and Voxel-Man® [6], which offer unlimited drilling training, in either a simulated environment or office setting. A notable limitation of these models is that the haptic feedback is dissimilar to real-life feedback; therefore, it is not possible to use these models for middle ear surgery training.

Artificial temporal bones offer advantages such as easy handling and the elimination of infection risk and the need for a training facility [4]. Registered artificial temporal bones for surgical practice, such as Phacon®, Biomodex®, and Otobone®, are widely available on the market [4]. The vast majority of commercially sold artificial temporal bones and artificial bones developed by researchers are based on cone-beam computed tomography (CBCT) [7] or computed tomography (CT) [2, 8–12] images. Sieber et al. [13] found that while CBCT is a functional method for imaging patients' ears, artifacts can be observed in small areas of the imaged ear. Similarly, a CBCT-based temporal bone model generated by Frithioff et al. [7] also exhibited failure in the middle ear area during the printing phase. According to Mukherjee's review [3], CT data have not been sufficient for detailed modeling of the ossicles and soft tissues in the middle ear.

High-resolution micro-CT images can be used to reduce the challenges associated with traditional CT data [3]. Phacon® was developed based on micro-CT image data from human temporal bones [1]. According to Chien et al. [1], Phacon® offers a limited number of different models, which curtails the possibility of surgical training. Additionally, Probst et al. [14] found that in the Phacon® model, ossicle mobility is greatly reduced, suggesting that achieving realistic ossiculoplasty training would be difficult using this model.

Skratz et al. [2] created a 3D-printed model based on CT imaging but emphasized the benefits of micro-CT for obtaining more detailed anatomical information. Rose et al. [15] compared both CT-based and micro-CT-based 3D-printed temporal bones. Notably, the model based on micro-CT imaging represented the middle ear structures more accurately.

Chien et al. [1] highlighted the strong anatomical resemblance between 3D-printed models and cadaveric temporal bones. Frithioff et al. [7] reported that printed models can act as supplementary aids during cadaveric training programs. Furthermore, Yuan et al. [16] stressed the importance of the repetitive training allowed by 3D-printed models, which enables novice surgeons to gain confidence and experience in their surgical specialty. However, they noted that, historically, the hidden costs associated with 3D printing have reduced its usability for educational purposes. Jenks et al. [10] and Stramiello et al. [9] developed a 3D-printed model specifically for training in endoscopic ear surgery. Stramiello's model is designed to be easily modifiable to simulate various middle ear conditions and is intended to be further developed for ossiculoplasty training [9]. Overall, researchers working on this topic support the incorporation of 3D-printed models in training programs.

Ossiculoplasty requires advanced microsurgical skills, but no published studies have yet examined the feasibility of 3D-printed temporal bone models for ossiculoplasty training. Ossiculoplasty simulation requires precise resolution due to the small size and high sensitivity of the ossicles. Bakhos et al. [17] created a 3D-printed temporal bone model, which was primarily intended for use in mastoid surgery simulation but included the application of two different active middle ear implants. Mukherjee et al. [18] explored possibilities for detailed imaging and 3D printing of the temporal bone to enable customized prosthetic solutions for the middle ear. Their study showed that 3D-printing techniques were needed to obtain accurate model micro-CT images and vat photopolymerization.

This study focuses on the usability of 3D-printed middle ear models in otosurgical training. It is based on our previous research, in which we manufactured a 3D-printed partial ossicular replacement prosthesis (PORP) [19]. We use a 3D-printed PORP alongside a 3D-printed middle ear model in our surgical simulation because the 3D-printed PORP has been shown to be comparable to a titanium equivalent but is much cheaper to manufacture and can therefore be used for teaching and simulations. Our aim was to utilize micro-CT imaging to obtain accurate representations of the middle ear structures. Ossiculoplasty was simulated by otosurgeons and otolaryngology–head and neck surgery (ORL-HNS) residents.

## Materials and methods

### Ethics and permissions

The study fulfilled the Helsinki Declaration for the ethical use of human material. The Institutional Review Board at Helsinki University Hospital approved the study protocol and the use of anonymous cadaveric temporal bones (Approval No. §49/29.10.2020, HUS/58/2020). The temporal bone was dissected at the Department of Forensic Medicine, University of Helsinki, with the permission of the National Supervisory Authority for Welfare and Health (Permission No. 6834/06.01.03.01/2013).

All the participants gave their informed consent to participate in the study.

### Micro-CT imaging

To generate a realistic model, a cadaveric temporal bone was imaged using a micro-CT system (GE, Phoenix v|tome|xs, Wunstorf, Germany) with a resolution of 18.8 microns. A total of 2,700 angles were used during the imaging process, with each angle having an expected exposure time of 500 ms. The average of the three exposures was calculated. The acquired micro-CT data were processed using Thermo Fisher PerGeos 2020.2 software (Waltham, Massachusetts, United States). The data were subjected to 2-phase watershed segmentation, and the resulting segmented data were converted into a surface representation. Finally, the surface model was exported as a stereolithography (STL) file, which was ready for further utilization and analysis. Only bony structures were modeled.

### 3D modeling and printing

The first phantom prototype was printed from the original STL file without any modeling. The material used was acrylonitrile butadiene styrene (ABS) Plus, which is white in color. The 3D printer used was a material extrusion-based uPrint SE Plus (Stratasys, Ltd., Eden Prairie, Minnesota) with a layer thickness of 0.254 mm. ABS Plus was deemed unsuitable for the middle ear phantom. Detailed reasons for this decision are provided in the Results section. Consequently, a different material was used to produce the second prototype.

**Fig. 1:**
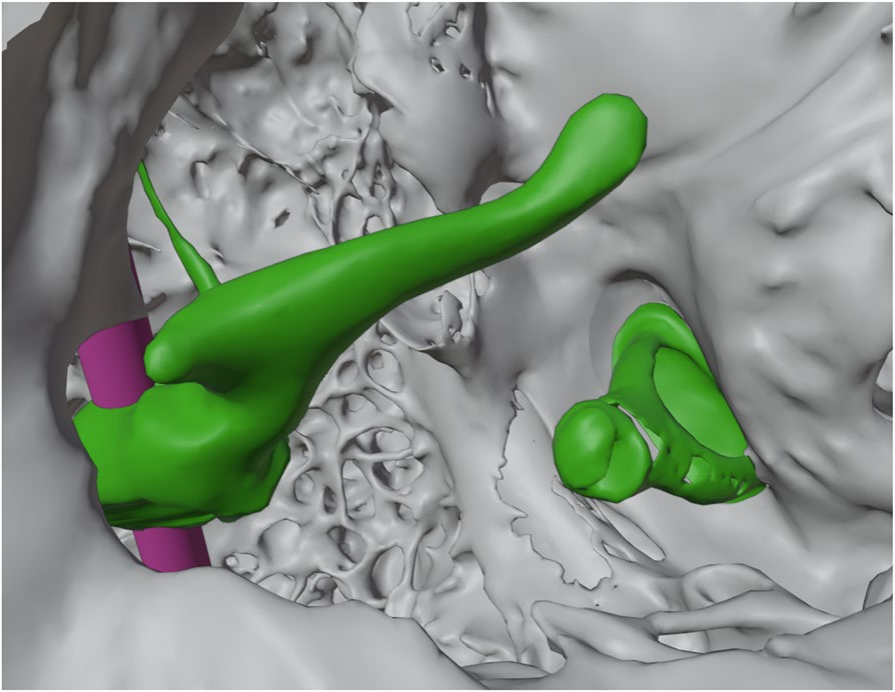
Three-dimensional (3D) model of the middle ear of the phantom. The support structure (pink) keeps the malleus (green) in the correct position

**Fig. 2:**
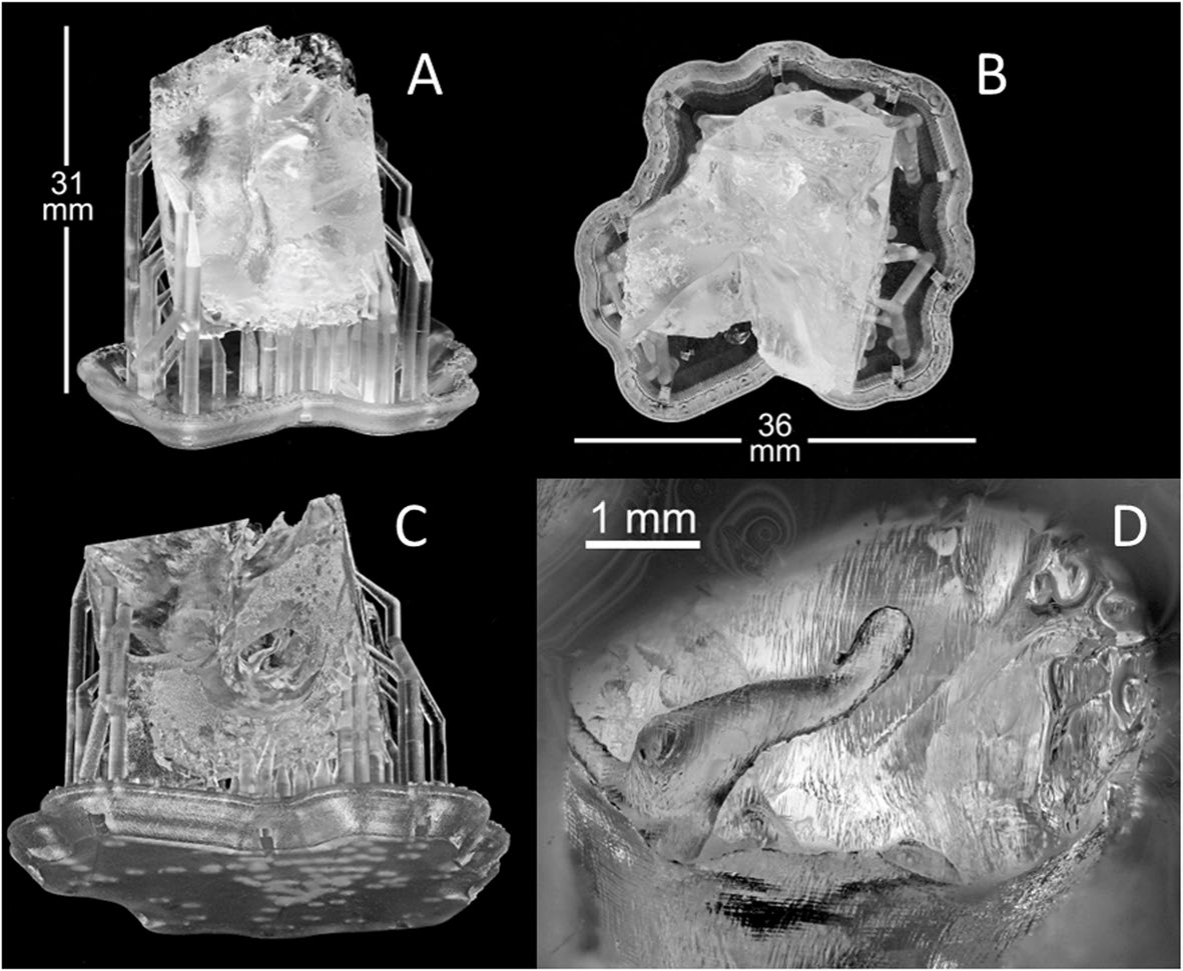
Three-dimensionally (3D) printed phantom (macro photography by M.Sc. (Tech) Pekka Paavola) from the anterior (**A**), proximal (**B**), and inferior (**C**) views. A magnified view of the canal showing the structures in the middle ear, such as the malleus and the stapes head (**D**)

In the 3D modeling phase of the production of the second phantom prototype, the incus was removed using 3D printing data preparation software (3Data Expert, DeskArtes Ltd., Helsinki, Finland). Figure 1 shows a 3D model of the support structure in the middle ear cavity. The supporting rod was automatically added to the print preparation software (Preform 3.9.0; Formlabs Inc., Somerville, MA) to ensure that the malleus was in the correct position. The software (Preform 3.9.0) also added support structures (shown in Fig. 2) to ensure that the phantom was attached to the printing platform. The configuration of the PORP was modeled from a commercial titanium PORP (MNP Malleus Notch Partial Prosthesis, Heinz Kurz GmbH, Dusslingen, Germany). The phantom and the prosthesis underwent the same laser-based vat photopolymerization printing process. The liquid photopolymer Clear V4 (Formlabs Inc.) was used as the material for both the middle ear phantom and the PORP. The chosen layer thickness was 25 μm, the laser spot size was 85 μm, and the XY resolution was 25 μm. After printing, FormWash (Formlabs Inc.) was used to clean the parts with pure isopropanol for 10 min. The parts were then cured in FormCure (Formlabs Inc.) for 15 min at 60°C. [19] The external support structures were removed from the phantom to allow the surgeons to access the external ear canal. The support structure of the malleus was left in place.

### Surgical simulation

The phantom was evaluated by ten otosurgeons and ten ORL-HNS residents. All the otosurgeons worked in university hospitals as otologists, had extensive experience in middle ear surgery, and taught in temporal bone courses. The residents were participants in a national temporal bone course and had 2–5 years’ experience in ORL-HNS. All the residents had practiced middle ear and mastoid surgery with cadaveric temporal bones during the course. Before enrolling in the course, eight of the ten residents had trained in otosurgery with cadaveric temporal bones.

**Fig. 3:**
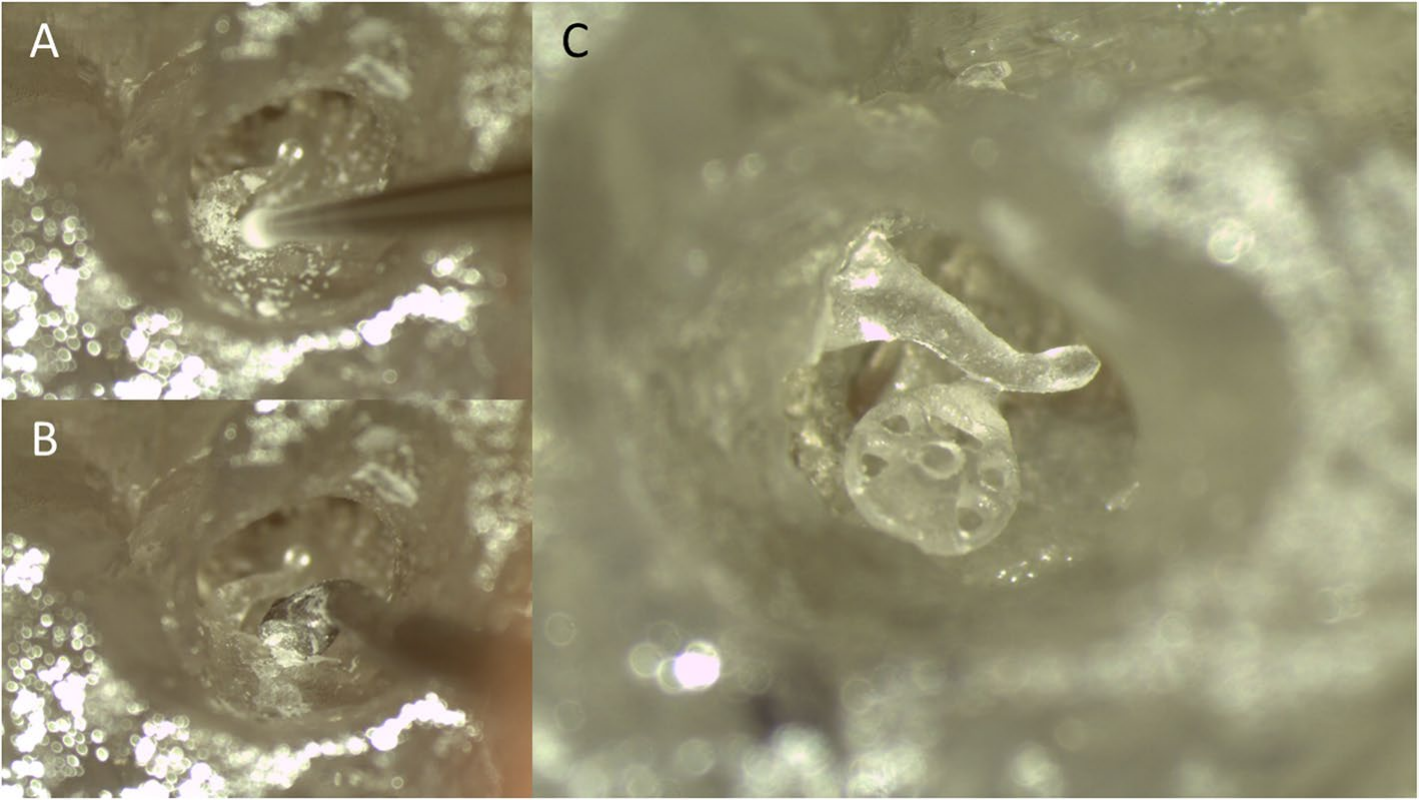
The simulation included drilling (**A**), scooping (**B**), and inserting the three-dimensionally (3D) printed partial ossicular replacement prosthesis (PORP) into the phantom (**C**). A video recording of the steps of the simulation is available in Additional File 1

To test the surgical usability of the 3D-printed models, an ossiculoplasty simulation was organized. In the simulation, the participants had to identify predefined anatomical landmarks of the middle ear. The phantom contained only bony structures; thus, there was no tympanic membrane. Once the anatomical landmarks had been established, the participants started ossiculoplasty. First, they drilled (Stryker, 5400–50 Core, Kalamazoo, MI) and scooped the back wall of the external auditory canal to visualize the stapes and its footplate. Subsequently, the participants manually inspected the movement of the malleus and stapes. Then, a 3D-printed PORP of 2.25 mm length was inserted between the malleus handle and the stapes head. The optimal length of the PORP was evaluated before simulation. At the end of the simulation, the participants inspected the movement of the PORP and stapes when manipulating the handle of the malleus. The steps of the simulation are shown in Fig. 3 and Additional File 1.

### Questionnaire

Once the participants had completed the simulation, they filled in the questionnaire. The questionnaire included statements about the participants’ experiences, and the participants responded on a scale with the options of agree, neutral, and disagree. Slightly different questionnaires were used for otosurgeons and ORL-HNS residents. Otosurgeons responded to 14 statements, which included more detailed statements about the PORP insertion: specifically, the 3D-printed PORP was compared to commercial PORPs, and respondents were asked about future training opportunities and needs. The ORL-HNS residents responded to eight statements. Both groups’ questionnaires also included a free comment section. The full questionnaires, including all the responses, are available in Additional File 2.

## Results

### Middle ear phantom

For the initial 3D printing, a rigid white material (ABS Plus) was used and subsequently analyzed microscopically. The printing process produced a detailed middle ear model, in which it was possible to identify the middle ear anatomical landmarks, including the ossicular chain. However, due to the material’s texture, the ossicular chain was rigid and inflexible. Removing the incus caused the malleus to lose its support, and the malleus was also detached. The remaining stapes were completely immobile. To overcome these obstacles, we changed the printing material to the transparent liquid photopolymer Clear V4, which we used in our previous study. [19] Additionally, to allow PORP surgery, the incus had to be removed from the model. To hold the malleus in place, an additional supportive rod for the malleus was 3D modeled (Fig. 1) and printed.

Use of the liquid photopolymer Clear V4 allowed superior printing resolution. The supportive rod kept the malleus in place and allowed minor flexible movements. Likewise, the stapes superstructure became flexible as well. For these reasons, transparent material was chosen for the creation of the final phantom (Fig. 2).

### Ossiculoplasty simulation by otosurgeons

**Fig. 4:**
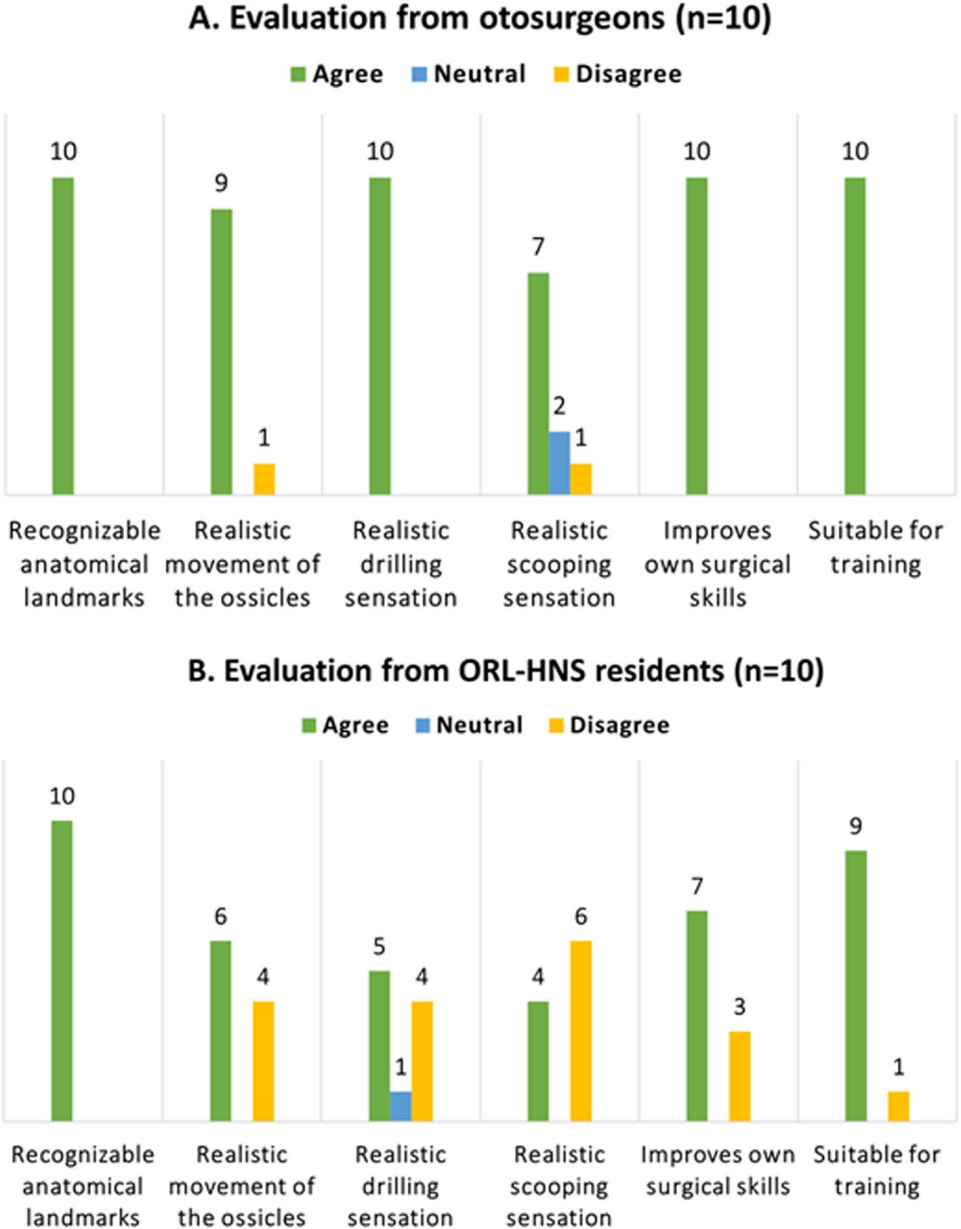
Evaluation by otosurgeons (**A**) and otolaryngology–head and neck surgery (ORL-HNS) residents (**B**). The evaluation scale included the options of agree, neutral, and disagree

The ossiculoplasty simulation was conducted in a temporal bone laboratory using an otologic microscope, high-speed drill system, and otologic instruments. Each otosurgeon had their own 3D-printed middle ear phantom and PORP available. The participants performed the simulation and evaluation individually and at their own pace. The responses of the otosurgeons are summarized in Fig. 4A. All the questions and answers can be found in Additional File 2. All the otosurgeons agreed that the anatomical landmarks were recognizable in the phantom. Nine out of the ten otosurgeons agreed that the phantom’s ossicular chain movement without incus was realistic, and only one disagreed. All the otosurgeons agreed that the drilling sensation was comparable to that created by a real temporal bone. However, when discussing the comparability of the scooping sensation, seven out of ten otosurgeons agreed that the phantom was comparable to a temporal bone, two otosurgeons remained neutral, and one disagreed. All the otosurgeons agreed that the structure and shape of the 3D-printed PORP corresponded to the conventional titanium PORP currently used in PORP ossiculoplasty. In terms of the maneuverability of the PORP into the phantom, eight out of the ten otosurgeons agreed that the 3D-printed PORP was comparable to the titanium PORP. However, one otosurgeon was neutral, and one disagreed with the statement. Overall, all the otosurgeons agreed that the simulation enabled them to improve their skills and that they could use 3D-printed prostheses for their own training in the future. All the otosurgeons thought that the simulation would be suitable for microsurgical training of residents and specialists. Eight out of ten identified the need for personalized patient phantoms when planning surgeries in the future. Opinions on the need for PORPs and total ossicular replacement prostheses (TORPs) varied in the questionnaires. Half of the surgeons agreed that there is a need for personalized prostheses in actual surgical procedures.

### Ossiculoplasty simulation by ORL-HNS residents

The results from the ORL-HNS residents are shown in Fig. 4B. Regarding the middle ear phantom, six out of the ten residents reported that the movement of the ossicles in the phantom corresponded to the movement of the ossicles in the temporal bone of the cadaveric. Half of the residents agreed that the drilling sensation was comparable to that created by the cadaveric temporal bone. Four out of the ten residents considered the scooping sensation comparable to that experienced when working with a cadaveric, while six disagreed. Regarding the 3D-printed PORPs, nine out of the ten residents managed to place the PORP prosthesis in its correct position in the phantom. The residents identified a need for future use of 3D-printed phantoms and prostheses for training purposes. Seven out of the ten residents thought that the use of 3D-printed phantoms enabled them to improve their surgical skills and was just as useful as working on cadaveric bones. However, only two residents agreed that the simulation was similar to a real-life scenario and that it improved their manual dexterity. Additionally, only three out of the ten residents acknowledged the need to practice with 3D-printed phantoms rather than cadaveric temporal bones in the future.

## Discussion

Cadaveric temporal bones remain the gold standard in otosurgical training. However, due to the limited availability, cost, and potential infectious hazards [4] of cadavers, identifying novel ways to train future otosurgeons is of the utmost importance. Middle ear surgery training is especially challenging without cadaveric temporal bones. In this study, we tested the suitability of 3D-printed artificial middle ears for surgical ossiculoplasty simulation. Both the otosurgeons and ORL-HNS residents found the simulation realistic and useful for otosurgical training.

Micro-CT is effective for imaging the temporal bone when detailed information about the structures of the middle ear or inner ear is needed. In the STL file used in this study, the middle ear ossicles, the cochlea, and the semicircular canals of the inner ear were clearly visible. The strength of 3D modeling is that it allows for simulating different anatomical or pathological variations in any given STL model. The same model can be printed several times and can be used for training purposes. Using identical 3D-printed temporal bone models could prove to be advantageous, for instance in temporal bone courses.

The first middle ear model that was printed in this study used white ABS Plus material and proved unsatisfactory. The white material mimicked real bone both in color, providing realistic visual impressions, and in texture, providing realistic haptic feedback during drilling. However, a major disadvantage of the white material was that the resolution was lower than expected. ABS is commonly used material when planning surgery. The structures of the middle and inner ears require high resolution due to their hollow and flexible surfaces [3]. However, when using ABS Plus, some of the more intricate structures were lost into the phantom. This approach reduced the realism of the white phantom. Additionally, the material was stiff, and the ossicular chain broke during manipulation.

The material used in the second temporal bone prototype, photopolymer Clear V4, was initially more difficult to manipulate due to the reflection of light from the operating microscope. However, a benefit of Clear V4 is its flexibility, which allows it to mimic the movements of real ossicles upon manipulation. Due to Clear V4’s properties, it was possible to print the models at a higher resolution. The Clear V4 allows for a minimum layer thickness of 0.025 mm, whereas the white resin has a minimum layer thickness of 0.05 mm. A smaller layer thickness enables higher resolution in the Z-direction. Consequently, more complex structures and anatomical landmarks could be identified in this model than in the ABS model. Clear V4 is thus superior to white resin, as the aim of a phantom is to replicate the middle ear with maximal preservation of anatomy and function.

As noted, the otosurgeons and ORL-HNS residents agreed that landmarks such as the stapes and malleus were clearly identifiable in the middle ear. This consensus highlights that 3D models are generally accepted regardless of a surgeon’s level of experience and suggests that due to their ease of use, phantoms can be incorporated into otorhinolaryngology training programs. Furthermore, both otosurgeons and ORL-HNS residents identified the need for 3D-printed phantoms for either their own training or for planning surgeries. This result suggests that there is a need for both 3D-printed models and cadaveric dissections. Simulations with 3D-printed models can supplement the training programs currently available to otosurgeons. Interestingly, almost all the otosurgeons agreed that the scooping and drilling sensations produced by the 3D-printed model were comparable to those experienced when working with a cadaveric temporal bone, whereas only half of the ORL-HNS residents agreed. This discrepancy raises the question of whether these differing opinions are related to surgical experience.

A comparison of Fig. 4A, B reveals that the otosurgeons’ responses were much more homogenous than those of the ORL-HNS residents. ORL-HNS residents have less experience with real temporal bones; however, many of them still found the phantom handling and surgical procedures unrealistic. This belief may arise because ORL-HNS residents may be more pessimistic about practicing surgery on an artificial middle ear than on a real cadaveric ear. The residents may not want the temporal bone used in training to be replaced by artificial material, which was also reflected in their answers. It is also possible that transparent resin was more problematic for ORL-HNS residents than for otosurgeons.

In this study, we demonstrated that it is possible to print a haptically realistic middle ear model that can be used in ossiculoplasty practice. However, the quality of printing materials is the greatest challenge. With current 3D printers, it is possible to print different colors, as described by Jenks et al. [10], but the compatibility and durability of the materials when drilling should be considered. On the other hand, even in cadaveric bones, the color differences are reduced compared to those in living bones [8]. According to Gadaleta et al. [8], due to the likelihood of postmortem transformations in cadaveric temporal bone, ORL-HNS residents may struggle more than otosurgeons when searching for fundamental structures, as they may appear unfamiliar. In addition, the absence of contrasting colors in the soft tissues of cadaveric temporal bones may lead to difficulty in locating critical structures. The color difference between cadaveric temporal bones and living soft tissue may make this mode of teaching unproductive for novice ORL-HNS residents [8]. Additionally, removal of an untampered temporal bone from the rest of the calvaria may damage the delicate structures inside the temporal bone. Henceforth, printed models may meet the demand in regard to quantity but also provide a more durable option than cadaveric bones [2].

Chenebaux et al. [20] used white resin as the material for a temporal bone prototype, but based on their evaluation, the dust from drilling was too heavy and stuck in the suction tube. They concluded that the structure of the resin needed to be of higher quality. The resin they used has been developed to mimic ABS. Our survey did not consider the composition of the drilling dust, but the otosurgeons did not complain about it. On the other hand, drilling was rather limited in our ossiculoplasty simulation. In addition, in plastic structures such as this one, the possibility of drilling dust getting into the eyes or respiratory system should be considered. Rose et al. [15] took this into account in their own evaluation but received no feedback on the adverse effects. The safety of these materials must be considered in the future.

In the future, otosurgical training and surgical planning could use patients’ high-resolution CT scans to construct personalized phantoms. This practice may encourage surgeons to approach complex cases by anticipating and evaluating any surgical risks that may arise, in turn minimizing the chance of surgical error [21]. Integrating 3D temporal bone training simulations into the curriculum will enhance the quality of surgical training [8]. In relation to resident training, Frithioff et al. [7] suggested that it may be more valuable to analyze the phantom’s effect on learning than to produce a high-quality phantom that is an exact replica of a temporal bone. In contrast, the needs of experienced surgeons may differ because phantoms should resemble the patient's anatomy to enable planning for procedures [7]. The approach and materials used in this study could serve the needs of both ORL-HNS residents and otosurgeons.

## Conclusion

Our simulated surgical intervention involved a complex procedure, ossiculoplasty, that can be practiced in a controlled and safe setting without the need to spend time on aseptic techniques. Furthermore, the participants’ opinions aligned with the findings of previous studies, in that they were in favor of implementing 3D-printed models in the otosurgical curriculum and surgical planning. There is consensus that this middle ear model is suitable for ossiculoplasty practice with regular commercial prostheses or with 3D-printed practice prostheses. In the future, it would be beneficial to develop a functional middle ear model. This model could be used both to practice the placement of bespoke middle ear prostheses and to measure how well the prostheses are positioned. Laser Doppler vibrometry could be used to assess prostheses' position and whether they transmit vibrations correctly.

### Supplementary Information

**Additional file 1:** Ossiculoplasty simulation. Simulation video of drilling, scooping, and placing a 3D-printed prosthesis (PORP) in the 3D-printed middle ear

**Additional file 2:** Responses of otosurgeons and ORL-HNS residents to the simulation questionnaire

## Data Availability

The collected data used during the study are available in the supplementary information files. The online version contains supplementary material available at https://doi.org/10.5281/zenodo.10370071.

## Abbreviations

3D: Three-dimensional
CBCT: Cone-beam computed tomography
CT: Computed tomography
ORL-HNS: Otolaryngology–head and neck surgery
PORP: Partial ossicular replacement prosthesis
STL: Stereolithography
TORP: Total ossicular replacement prosthesis
