# Supplementary material for: Integration of 3D-printed middle ear models and middle ear prostheses in otosurgical training
Source: BMC Med Educ. 2024 Apr 24;24:451. doi: 10.1186/s12909-024-05436-9 (PMC11044351; doi:10.1186/s12909-024-05436-9)
Supplement: Supplementary file 2 — Additional file 2: Responses of otosurgeons and ORL-HNS residents to the simulation questionnaire [file 12909_2024_5436_MOESM2_ESM.pdf]

Evaluation from otosurgeons (n=10)	Agree	Neutral	Disagree
1. Anatomical landmarks are recognizable in the middle ear phantom.	10	0	0
2. The movement of ossicles within the phantom correspond to the movement of the ossicles in a real middle ear.	9	0	1
3. The drilling sensation is comparable to a temporal bone.	10	0	0
4. The scooping sensation is comparable to a temporal bone.	7	2	1
5. The 3D-printed PORP corresponds in structure/shape to a titanium PORP.	10	0	0
6. The maneuverability of the 3D printed PORP is comparable to a titanium PORP.	8	1	1
7. Inserting a 3D printed PORP into a phantom can be compared to inserting a titanium PORP into the middle ear.	8	1	1
8. This type of simulation allows me to improve my surgical skills.	10	0	0
9. In the future, I would like to use the phantom and 3D printed middle ear prostheses for my own training.	10	0	0
10. This type of simulation is suitable for the training of specialists in otologic surgery.	10	0	0
11. This type of simulation is suitable for otologic surgery for residents (e.g., temporal bone drilling course).	10	0	0
12. In the future, personalized phantoms corresponding to patients will be required in the planning of surgeries.	8	1	1
13. In the future, there will be a need for personalized 3D printed PORPs in patient surgeries.	5	4	1
14. In the future, there will be a need for personalized 3D printed TORPs in patient surgeries.	5	4	1

Evaluation from ORL-HNS residents (n=10)	Agree	Neutral	Disagree
1. The ossicles (malleus and stapes) can be clearly found in the phantom.	10	0	0
2. The movement of the phantom's ossicles corresponds to the movement of real ossicles.	6	0	4
3. The drilling sensation is comparable to a temporal bone.	5	1	4
4. The scooping sensation is comparable to a temporal bone.	4	0	6
5. I managed to set the PORP prosthesis into the phantom.	9	0	1
6. Practicing with a 3D printed phantom allows me to improve my surgical skills just as good as practicing with cadaver bones.	7	0	3
7. In the future, I would rather practice with 3D printed phantoms than cadaver bones.	3	2	5
8. In the future, I would like to use 3D printed phantoms and middle ear prostheses for my own training.	9	0	1
